# Survival benefit of perioperative chemoradiotherapy in patients with resectable primary gastric adenosquamous carcinoma: a population-based cohort study

**DOI:** 10.3389/fonc.2025.1540106

**Published:** 2025-07-02

**Authors:** Di Wu, Jie Li, Bai-Shu Dai, Qi-Ying Song, Peng Chen, Ming-Yu Gu, Bing-He Zhao, Lu Liu, Zheng-Yao Chang, Wen-Xing Gao, Wen Zhao, Shuo Li, De-Bin Zheng, Tian-Yu Xie, Xin-Xin Wang, Lin Chen

**Affiliations:** ^1^ Department of General Surgery, First Medical Center of Chinese People's Liberation Army (PLA) General Hospital, Beijing, China; ^2^ Medical School of Chinese People's Liberation Army (PLA), Beijing, China; ^3^ School of Medicine, Nankai University, Tianjin, China; ^4^ Medical Innovation Research Division of Chinese People's Liberation Army (PLA) General Hospital, Beijing, China

**Keywords:** gastric adenosquamous carcinoma, gastric cancer, chemoradiotherapy, chemotherapy, radiotherapy, survival benefit

## Abstract

**Background:**

Currently, whether Gastric adenosquamous carcinoma (GASC) can benefit from perioperative chemoradiotherapy (pCRT) remains controversial. The objective of this study was to evaluate the survival benefit of pCRT in resectable GASC.

**Methods:**

Patients diagnosed with GASC were selected from the Surveillance, Epidemiology, and End Results (SEER) Program and our medical center. Enrolled patients were stratified into two cohorts according to whether they underwent perioperative chemotherapy and radiotherapy or not, and the overall survival (OS) and cancer-specific survival (CSS) of the two cohorts were compared. Subsequent subgroup analysis was performed to identify the population that could demonstrably benefit from chemoradiotherapy.

**Results:**

We screened almost 180,000 cases of gastric malignant neoplasms. Finally, only 267 patients with GASC met the inclusion criteria and were eventually included in the study, with 147 and 120 patients in the pCRT and non-pCRT groups, respectively. The baseline information of the two groups showed no statistically significant differences. Patients in the pCRT group had superior OS (26.0 vs. 13.0 months, p=0.002) and CSS (26.0 vs. 14.0 months, p=0.004). Univariate and multivariate COX regression analyses demonstrated that pCRT was an independent protective factor for favorable OS and CSS with in patients with GASC and age, race, tumor size, T stage, N stage and TNM stage were also independent predictors of survival. Subgroup analysis indicated that the GASC population aged ≤ 66 years, non-EGJ, tumor > 5 cm, tumor differentiation degree 3-4, T3-4 stage, N2-3, and TNM III-IV could significantly benefit from pCRT. The combined chemotherapy with radiotherapy group significantly improved OS and CSS of GASC compared to chemotherapy alone.

**Conclusions:**

This retrospective study verified that pCRT could improve the long-term OS and CSS of patients with GASC. Subgroup analysis found that patients with aged ≤ 66 years, tumor differentiation grade 3-4, T3-4, N2-3, and TNM III-IV could gain significant benefits from perioperative chemoradiotherapy. Moreover, the study demonstrated that patients with GASC receiving combined radiotherapy and chemotherapy had superior OS and CSS compared to those receiving chemotherapy alone, implying the crucial role of radiotherapy. This study provides an excellent evidence-based medical reference for GASC treatment.

## Introduction

Gastric cancer (GC) is one of the common malignant tumors and also a leading cause of cancer-related deaths worldwide (1). Gastric adenosquamous carcinoma (GASC) is a relatively rare pathological type of gastric cancer that simultaneously contains adenocarcinoma and squamous cell carcinoma components, accounting for approximately 1% of the overall incidence of gastric cancer (2–5). However, the majority of current studies have demonstrated that GASC exhibits a more malignant biological behavior, stronger invasiveness and poorer survival prognosis than gastric adenocarcinoma (GAC) (6–9). In recent years, perioperative chemoradiotherapy has emerged as a crucial therapeutic modality for improving survival outcomes in GAC (10, 11). For locally advanced GAC, preoperative neoadjuvant chemoradiotherapy demonstrates significant clinical value by inducing tumor regression and downstaging, thereby increasing the likelihood of achieving R0 resection (12–14). Postoperative adjuvant chemoradiotherapy further contributes to long-term prognosis by eliminating minimal residual disease (MRD) and circulating tumor cells (CTCs), effectively reducing recurrence risks (15, 16).

Previous studies based SEER database analyses investigating perioperative chemoradiotherapy in gastric cancer have yielded important insights. A study by Che et al. revealed that perioperative chemoradiotherapy (PCRT) significantly improved survival in gastric cancer patients with stage III, diffuse-type histology, tumors >34 mm, or lymph node-positive status (17). Additionally, Yeh et al. reported postoperative chemoradiotherapy (CRT) conferred survival benefits in elderly patients (65–79 years) with stage II-III gastric cancer (18). Notably, neoadjuvant radiotherapy demonstrated particular efficacy in gastroesophageal junction adenocarcinoma (GEJA), achieving a 5-year overall survival (OS) rate of 38.2% in T3–4N+ tumors and intestinal-type subtypes (19). Currently, the therapeutic paradigm for gastric adenosquamous carcinoma (GASC) largely mirrors that of gastric adenocarcinoma. While some studies suggest improved overall survival (OS) with chemotherapy in GASC (2), others report no survival benefit from postoperative adjuvant chemotherapy (20). Given the aggressive biological behavior and higher malignant potential of GASC compared to conventional adenocarcinoma, the role of perioperative chemoradiotherapy in this rare subtype remains uncertain, with limited evidence available.

To address this knowledge gap, this study leveraged a dual data source approach, integrating SEER database records with institutional data from our medical center spanning two decades. By analyzing one of the largest GASC cohorts to date, we aimed to retrospectively evaluate the impact of perioperative chemoradiotherapy on long-term prognosis in this understudied population. This comprehensive analysis provides critical insights into optimizing therapeutic strategies for GASC patients.

## Methods

### Data source and patient selection

Patients with GASC in this study were obtained from Surveillance, Epidemiology, and End Results (SEER) Program (www.seer.cancer.gov) and Chinese People’s Liberation Army General Hospital (PLAGH). Our study utilized the SEER*Stat Database: Incidence-SEER Research Data, 17 Registries, Nov 2023 Sub (2000-2021)-Linked To County Attributes-Time Dependent (1990-2022) Income/Rurality, 1969-2022 Counties, National Cancer Institute, DCCPS, Surveillance Research Program, released April 2024, based on the November 2023 submission. Patients diagnosed with gastric cancer (GC) by histopathology were selected for this study between 2004 and 2023 from Chinese PLAGH according to the Ethics Committee of our institute. The study was conducted in compliance with the 1964 Helsinki Declaration, and patient informed consent was waived in this study because public data from SEER were available for secondary research and our hospital data were derived from medical records obtained from previous clinical treatments. In addition, the privacy and personally identifiable information of patients were protected according to the Ethics Committee of our institute.

**Figure 1:**
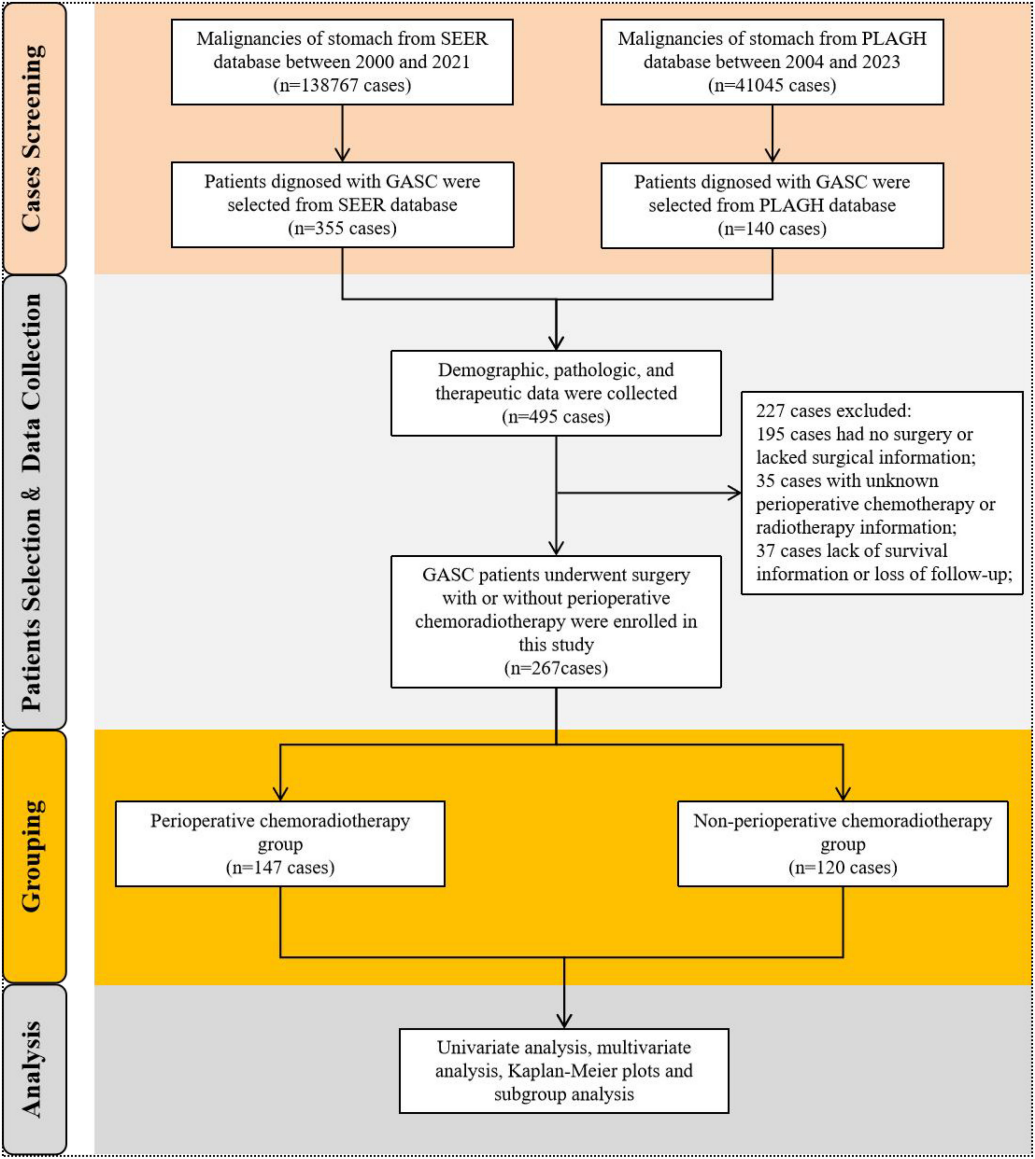
Flowchart of this study.

We accessed all available data on stomach malignancy diagnosed by histology for research in the SEER database. Above all, patients with ICD-O-3: 8560/3 (The International Classification of Diseases for Oncology, ICD-O-3), which was diagnosed with gastric adenosquamous carcinoma by positive histology, were selected as the focus of study. In addition, patients with GASC diagnosed by histopathology from our hospital center were enrolled in the study. Notably, cases in our institutional cohort were meticulously re-reviewed by a panel of experienced pathologists through a double-blind diagnostic process, with discrepancies resolved by a third independent expert, thereby ensuring standardized histopathological classification. And then, we formed the research population by excluding patients with GASC who had not undergone surgery, had no perioperative chemoradiotherapy records, or had incomplete survival information. Finally, GASC patients underwent perioperative chemotherapy and/or radiotherapy were enrolled into the pCRT group, and patients who did not undergo any perioperative chemotherapy or radiotherapy were included the Non-pCRT group. The selection flowchart was illustrated in **Figure 1**.

### Data collection

In order to extract the valuable variables and build a study database of GASC, we collected information from the SEER database and our hospital’s dataset respectively. The following information was gathered from SEER database: Age, Sex, Year of diagnosis, Race, Site code, Primary site, Grade, Derived EOD 2018 T (2018+), Derived EOD 2018 N (2018+), Derived EOD 2018 M (2018+), Derived EOD 2018 Stage Group (2018+), Derived AJCC T, 7th ed (2010-2015), Derived AJCC N, 7th ed (2010-2015), Derived AJCC M, 7th ed (2010-2015), Derived AJCC Stage Group, 7th ed (2010-2015), 7th Edition Stage Group Recode (2016-2017), Derived SEER Combined T (2016-2017), Derived SEERCombined N (2016-2017), Derived SEER Combined M (2016-2017), Derived SEER Cmb Stg Grp (2016-2017), Derived AJCC T, 6th ed (2004-2015), Derived AJCC N, 6th ed (2004-2015), CS mets at dx (2004-2015), Derived AJCC M, 6th ed (2004-2015), Derived AJCC Stage Group, 6th ed (2004-2015), RX Summ–Surg Prim Site (1998+), RX Summ–Scope Reg LN Sur (2003+), Radiation recode, RX Summ–Surg/Rad Seq, Chemotherapy recode (yes, no/unk), RX Summ–Systemic/Sur Seq, EOD Primary Tumor (2018+), EOD Regional Nodes (2018+), EOD Mets (2018+), Tumor Size Summary (2016+), Tumor Size Over Time Recode (1988+), Regional nodes examined (1988+), Regional nodes positive (1988+), CS tumor size (2004-2015), CS lymph nodes (2004-2015), CS extension (2004-2015), CS mets at dx (2004-2015), SEER Combined Mets at DX-bone (2010 +), SEER Combined Mets at DX-brain (2010+), SEER Combined Mets at DX-liver (2010+), SEER Combined Mets at DX-lung (2010+), Mets at DX-Distant LN (2016+), Mets at DX-Other (2016+), CS mets at dx (2004-2015), CS Tumor Size/Ext Eval (2004-2015), CS Reg Node Eval (2004-2015), CS Mets Eval (2004-2015), SEER cause-specific death classification, SEER other cause of death classification, Survival months, Year of follow-up recode and Year of death recode. Data collected from our hospital database included sex, age, clinical diagnosis, pathological diagnosis, treatment approach, primary tumor site, tumor size, pathological grade, tumor invasion depth (T stage classification), lymph node metastasis number (N stage classification), distant metastasis (M stage classification), chemotherapy and radiotherapy, treatment regimens, cause of death classification, survival status and overall survival time. The last follow-up date in our hospital database was June 2024. Overall survival (OS) defined as the time between diagnosis by histology and the date of death or the last follow-up date. Cancer-specific survival (CSS) was defined as the time between diagnosis by histology and the date of cancer-related death or last follow-up. Tumor stage was eventually evaluated based on the above codes according to the 8th edition of the AJCC on Gastric Cancer TNM staging system.

### Statistical analysis

Categorical variables are described as percentage (n, %), and comparisons were performed using the chi-square test. Continuous variables are represented as mean ± standard deviation (SD), and comparisons were performed using t-test. All statistical analyses in this study were carried out using SPSS software (version 26.0; IBM Corporation, Armonk, New York, USA) and R software (version R4.4.1). The Kaplan-Meier curves were used to analyze OS and cancer-specific survival (CSS) and were compared using the log-rank test. The Cox proportional hazards regression model was used for univariate and multivariate analyses to identify prognostic indicators. We assessed multicollinearity among variables exhibiting significant differences in the univariate analysis using the variance inflation factor (VIF) method. Subsequently, all factors achieving a p-value < 0.05 in the univariate analysis were incorporated into the multivariable regression model to identify independent predictors. The significance level was set at α = 0.05 for the two-sided test. The p value less than 0.05 was deemed statistically significant.

## Results

### Demographic and clinicopathological characteristics of GASC cohorts

A total of 138,767 cases of gastric malignant neoplasms were screened from the SEER database. Among them, 355 patients who were pathologically confirmed as GASC were enrolled in this study. Concurrently, 140 patients diagnosed with GASC from the gastric cancer database consisting of 41,045 cases in the PLAGH center were selected and incorporated into the GASC dataset of this study, bringing the total number of cases to 495. The demographic, clinical, and treatment details of 495 patients with GASC were gathered. Subsequently, 195 patients who had not undergone surgical treatment, 35 patients lacking perioperative treatment-related information, and 37 patients with incomplete survival data or who were lost to follow-up were excluded. Ultimately, 267 patients were included in the final analysis. We analyzed the clinical characteristics of the GASC population included in this study. GASC was predominantly found in males, with a male incidence approximately threefold that of females. The median age at onset was 66 years (range 36-90 years). The incidence of esophagogastric junction (EGJ) involvement is relatively elevated. The median tumor size was 5 cm (0.4-18.5 cm). Approximately 76.8% of GASC tumors manifest as low or undifferentiated tumors. The proportion of patients with TNM stage III or higher was 64.4%, suggesting a relatively high malignancy of GASC. Subsequently, the patients were divided into two cohorts according to whether they received perioperative chemoradiotherapy or not, namely the perioperative chemoradiotherapy group (pCRT) and the non-perioperative chemoradiotherapy group (non-pCRT), to analyze the impact of perioperative chemoradiotherapy on the long-term prognosis of patients with GASC. Among them, 147 patients were included in the pCRT group, and 120 patients were included in the non-pCRT group (**Figure 1**). In the pCRT cohort, detailed perioperative chemotherapy regimens were available for 46 patients with GASC from the PLAGH dataset. Unfortunately, specific treatment protocols could not be retrieved from the SEER database. As shown in **Supplementary Table S1**, the majority of perioperative chemotherapy regimens for GASC consisted of taxane-based combinations with either fluoropyrimidines (e.g., 5-fluorouracil [5-FU]) or platinum analogs (27/46 patients). The remaining regimens involved the combination of fluoropyrimidines and platinum agents (19/46 patients). We compared the baseline information of the two groups of patients and found that there were no statistically significant differences between the two groups in terms of age, sex, ethnicity, tumor location, size, pathological grade, T stage, N stage, M stage, and TNM stage of the tumor (p > 0.05) (**Table 1**).

**Table 1:** Demographic and clinicopathological characteristics of GASC cohorts with or without perioperative chemoradiotherapy.

Variables	Total population (n=267) N (%)	Non-pCRT group (n=120)	pCRT group (n=147)	P value
		N (%)	N (%)	
Demographic				
Age (years)				0.145
≤ 66	140 (52.4)	57 (47.5)	83 (56.5)	
> 66	127 (47.6)	63 (52.5)	64 (43.5)	
Sex				0.572
Female	69 (25.8)	29 (24.2)	40 (27.2)	
Male	198 (74.2)	91 (75.8)	107 (72.8)	
Race				0.306
White	148 (55.4)	61 (50.8)	87 (59.2)	
API	92 (34.5)	44 (36.7)	48 (32.7)	
Black	27 (10.1)	15 (12.5)	12 (8.1)	
Clinical				
Tumor location				0.811
EGJ	149 (55.8)	66 (55.0)	83 (56.5)	
Non-EGJ	118 (44.2)	54 (45.0)	64 (43.5)	
Tumor size (cm)				0.735
≤ 5	126 (47.2)	58 (48.3)	68 (46.3)	
> 5	141 (52.8)	62 (51.7)	79 (53.7)	
Pathological				
Grade				0.483
Well or Moderately differentiated	62 (23.2)	32 (26.7)	30 (20.4)	
Poorly or Undifferentiated	205 (76.8)	88 (73.3)	117 (79.6)	
T stage classification				0.426
T1-2	64 (24.0)	26 (21.7)	38 (25.9)	
T3-4	203 (76.0)	94 (78.3)	109 (74.1)	
N stage classification				0.754
N0-1	143 (53.6)	63 (52.5)	80 (54.4)	
N2-3	124 (46.4)	57 (47.5)	67 (45.6)	
M stage classification				0.996
M0	247 (92.5)	111 (92.5)	136 (92.5)	
M1	20 (7.5)	9 (7.5)	11 (7.5)	
TNM stage				0.101
I	23 (8.6)	16 (13.3)	7 (4.8)	
II	72 (27.0)	31 (25.8)	41 (27.9)	
III	152 (56.9)	64 (53.4)	88 (59.9)	
IV	20 (7.5)	9 (7.5)	11 (7.4)	

GASC, Gastric Adenosquamous Carcinoma; Non-pCRT group, Non-perioperative Chemotherapy or Radiotherapy group; pCRT group, Perioperative Chemotherapy or Radiotherapy group; API, Asian or Pacific Islander; EGJ, Esophagogastric Junction; TNM stage, tumor-nodes-metastasis stage.

### The impact of perioperative chemoradiotherapy on survival prognosis of GASC

The median OS and CSS of the total population of resectable GASC included in this study were 19.0 months (95% confidence interval [CI]: 17 - 22) and 20.0 months (95%CI: 17.1 - 22.9), respectively. The 1-year, 3-year, and 5-year cumulative OS rates of the total GASC population were 64.8%, 29.3%, and 21.9%, respectively, and the CSS rates were 67.0%, 33.3%, and 27.5%, respectively (**Supplementary Figure S1**). Univariate and multivariate COX regression analyses demonstrated that API and Black ethnicity, tumor diameter greater than 5 cm, T3-4, N2-3, M1, as well as TNM III-IV stage and the absence of perioperative chemoradiotherapy were independent risk factors affecting OS and CSS of patients with GASC (**Table 2**). Conversely, perioperative chemoradiotherapy was an independent protective factor for favorable OS and CSS inpatients with GASC (p < 0.001, hazard ratio [HR] = 0.436; 95% CI, 0.324 - 0.588 for OS; p < 0.001, HR = 0.445; 95%CI, 0.323 - 0.614 for CSS).

**Table 2:** Univariate and multivariate Cox regression analyses for overall survival and cancer-specific survival.

Characteristics	Total (n)	Overall Survival				Cancer-Specific Survival			
		Univariate analysis		Multivariate analysis		Univariate analysis		Multivariate analysis	
		Hazard ratio (95% CI)	P value	Hazard ratio (95% CI)	P value	Hazard ratio (95% CI)	P value	Hazard ratio (95% CI)	P value
Age	267								
≤ 66	140	Reference				Reference			
> 66	127	1.299 (0.990 - 1.705)	0.059			1.209 (0.899 - 1.624)	0.209		
Sex	267								
Female	69	Reference				Reference			
Male	198	1.208 (0.882 - 1.655)	0.239			1.137 (0.810 - 1.596)	0.459		
Race	267								
White	148	Reference		Reference		Reference		Reference	
API	92	2.402 (1.550 - 3.723)	< 0.001	2.295 (1.462 - 3.602)	< 0.001	2.565 (1.617 - 4.068)	< 0.001	2.310 (1.440 - 3.706)	0.001
Black	27	1.170 (0.863 - 1.586)	0.313	0.705 (0.511 - 0.973)	0.033	1.164 (0.840 - 1.611)	0.362	0.712 (0.504 - 1.007)	0.055
Tumor location	267								
EGJ	149	Reference				Reference			
Non-EGJ	118	1.244 (0.924 - 1.673)	0.150			1.244 (0.924 - 1.673)	0.150		
Tumor size	267								
≤ 5	126	Reference		Reference		Reference		Reference	
> 5	141	2.140 (1.617 - 2.832)	< 0.001	1.790 (1.317 - 2.433)	< 0.001	2.335 (1.717 - 3.175)	< 0.001	1.925 (1.376 - 2.692)	< 0.001
Grade	267								
1-2	62	Reference				Reference			
3-4	205	0.980 (0.714 - 1.345)	0.902			0.952 (0.678 - 1.336)	0.777		
T classification	267								
T1-2	64	Reference		Reference		Reference		Reference	
T3-4	203	2.309 (1.638 - 3.256)	< 0.001	2.264 (1.548 - 3.311)	< 0.001	2.132 (1.465 - 3.104)	< 0.001	2.027 (1.337 - 3.072)	0.001
N classification	267								
N0-1	143	Reference		Reference		Reference		Reference	
N2-3	124	2.154 (1.628 - 2.849)	< 0.001	2.115 (1.566 - 2.856)	< 0.001	2.120 (1.569 - 2.866)	< 0.001	2.073 (1.502 - 2.861)	< 0.001
M classification	267								
M0	247	Reference		Reference		Reference		Reference	
M1	20	2.716 (1.662 - 4.438)	< 0.001	2.059 (1.231 - 3.443)	0.006	3.143 (1.914 - 5.160)	< 0.001	2.355 (1.401 - 3.959)	0.001
TNM stage^*^	267								
I	23	Reference				Reference			
II	72	1.875 (0.992 - 3.545)	0.053			1.536 (0.737 - 3.200)	0.252		
III	152	4.359 (2.371 - 8.014)	< 0.001			4.087 (2.052 - 8.137)	< 0.001		
IV	20	8.002 (3.786 - 16.915)	< 0.001			8.587 (3.814 - 19.335)	< 0.001		
Chemoradiotherapy	267								
Non-pCRT	120	Reference		Reference		Reference		Reference	
pCRT	147	0.661 (0.502 - 0.870)	0.003	0.436 (0.324 - 0.588)	< 0.001	0.650 (0.483 - 0.875)	0.005	0.445 (0.323 - 0.614)	< 0.001

*Collinearity diagnosis was performed on variables that showed significant differences in the univariate analysis by the variance inflation factor (VIF) method. TNM stage had collinearity with T/N/M classification after being included in the multivariate analysis, so it was not included in the multivariate analysis. CI, Confidence Interval; API, Asian or Pacific Islander; EGJ, Esophagogastric Junction; TNM stage, Tumor-Nodes-Metastasis stage; Non-pCRT group, Non-perioperative Chemotherapy or Radiotherapy group; pCRT group, Perioperative Chemotherapy or Radiotherapy group.

**Figure 2:**
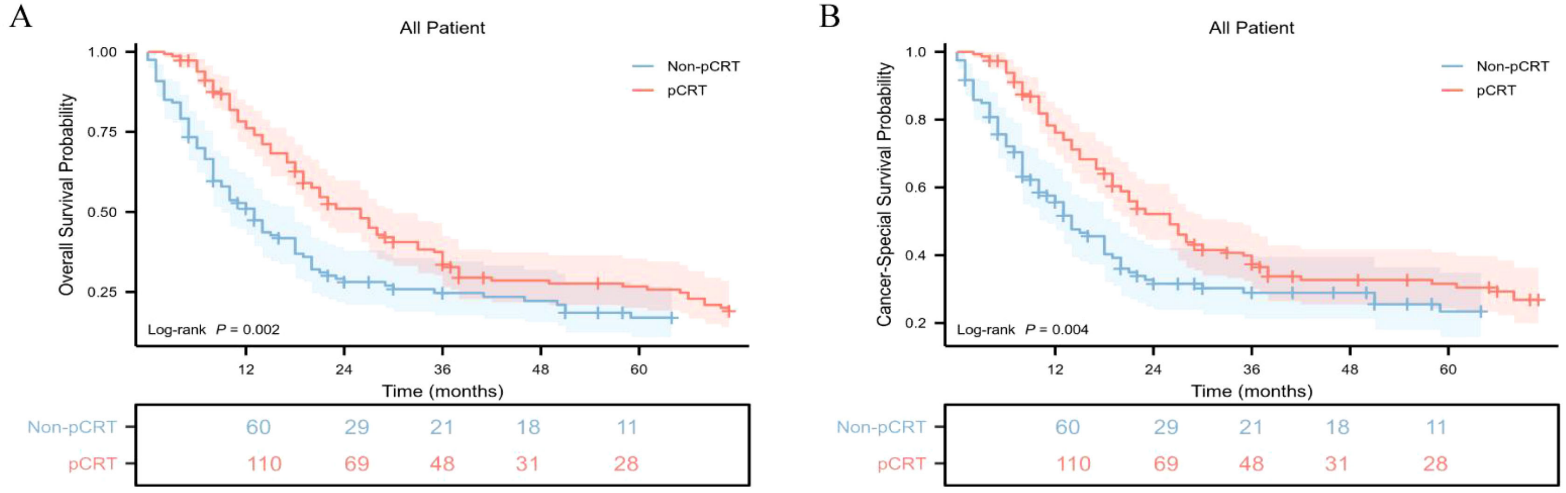
Kaplan-Meier survival curves of cumulative OS and CSS in different cohorts with gastric adenosquamous carcinoma in this study. **(A)** Kaplan-Meier survival curve of cumulative OS of the non-pCRT and pCRT cohorts; **(B)** Kaplan-Meier survival curve of cumulative CSS of the non-pCRT and pCRT cohorts.

Further analysis revealed that the median OS of the pCRT group was 26.0 months (95%CI: 20 - 30), which was significantly higher than that of the non-pCRT group, with a median survival time of 13.0 months (95%CI: 9 - 18), and the difference was statistically significant (p = 0.002) (**Figure 2A**). The 1-year, 3-year, and 5-year cumulative OS rates of the pCRT group were 76.1%, 33.6%, and 26.2%, respectively, whereas those of the non-pCRT group were 51.0%, 24.7%, and 17.0%, respectively. The median CSS of the two groups of patients was 26 months (95%CI: 21 - 33) and 14 months (95%CI: 11 - 19), respectively. The median CSS of the perioperative treatment group was significantly higher than that of the untreated group, and the difference was statistically significant (p = 0.004) (**Figure 2B**). The 1-year, 3-year, and 5-year CSS rates of the pCRT group were 76.1%, 37.4%, and 31.1%, respectively, while the 1-year, 3-year, and 5-year cumulative CSS rates of the non-pCRT group were 55.6%, 28.9%, and 23.4%, respectively.

### Subgroup interaction analysis

**Figure 3:**
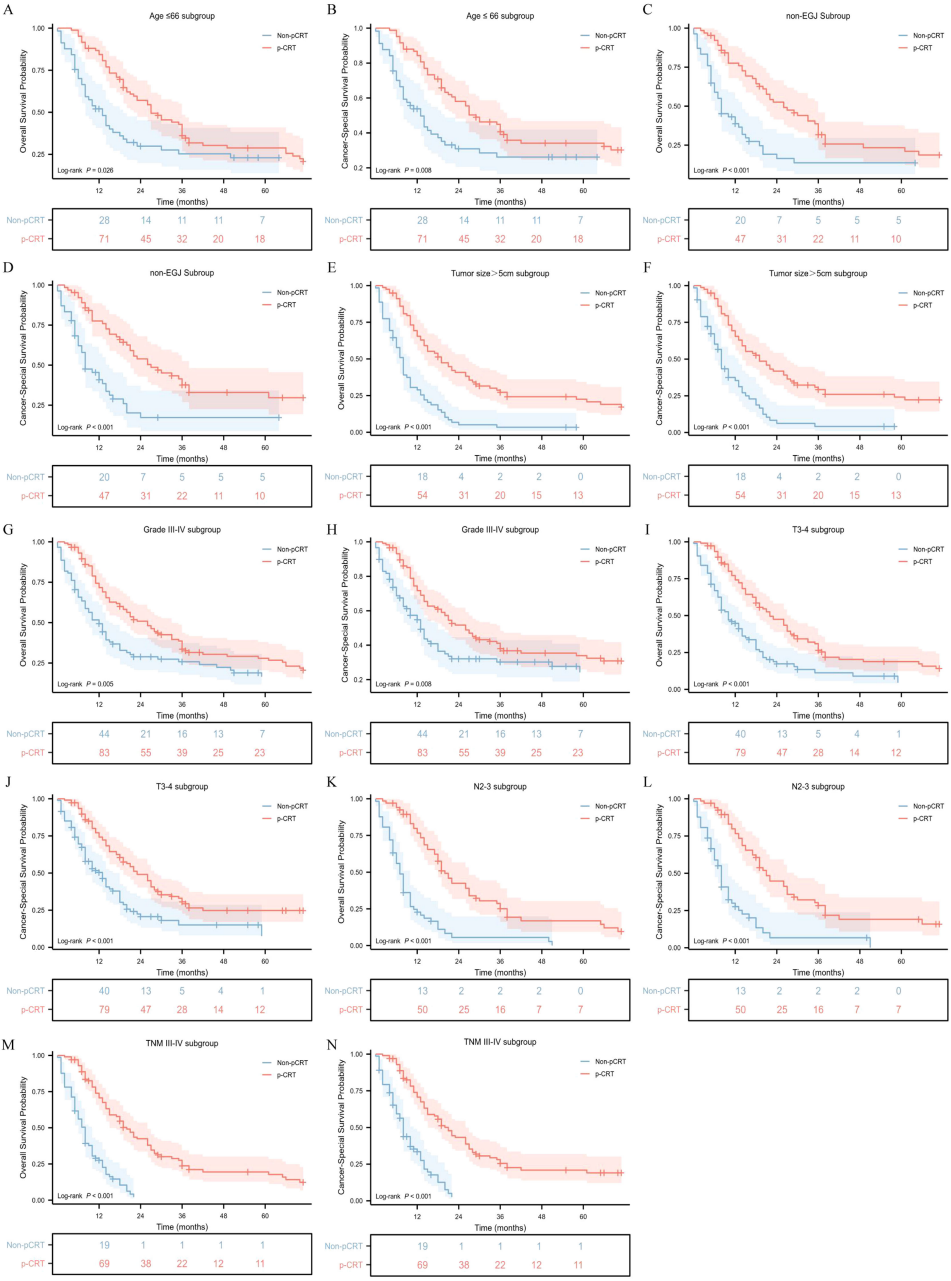
Overall survival and cancer-specific survival stratified by pCRT in different groups. Age ≤ 66 subgroup **(A, B)**, non-EGJ subgroup **(C, D)**, tumor size > 5cm subgroup **(E, F)**, Grade3-4 subgroup **(G, H)**, T 3-4 subgroup **(I, J)**, N 2-3 subgroup **(K, L)** and TNM III-IV subgroup **(M, N)**.

To identify the population that could benefit from perioperative chemoradiotherapy, a subgroup interaction analysis was conducted. We found that perioperative treatment significantly improved the OS and CSS of patients with GASC in the subgroup aged ≤ 66 years (p = 0.026, hazard ratio [HR] = 0.653; 95% confidence interval [CI], 0.431 - 0.988 for OS; p = 0.008, HR = 0.581; 95%CI, 0.372 - 0.907 for CSS) (**Figures 3A, B**). For the sex subgroup, although it was observed that pCRT seemed to significantly improve the median OS in male patients (p = 0.048, HR = 0.691; 95%CI, 0.466 - 1.023), the difference in its impact on the median CSS was not statistically significant (p = 0.056, HR = 0.675; 95%CI, 0.438 - 1.040) (**Supplementary Figures S2C–F**). When analyzing the relationship between ethnicity and pCRT for GASC, it was found that compared with White people (p = 0.165, HR = 0.772, 95%CI, 0.525 - 1.135), API and Black populations had a better response to pCRT, showing significant improvements in median OS and median CSS (p = 0.032, HR = 0.624; 95%CI, 0.383 - 1.015 for OS and p = 0.004, HR = 0.506; 95%CI, 0.299 - 0.855 for CSS in the API subgroup; p = 0.005, HR = 0.381; 95%CI, 0.165 - 0.879 for OS and p = 0.016, HR = 0.417; 95%CI, 0.173 - 1.008 for CSS in the Black subgroup) (**Supplementary Figures S2G–L**). Different tumor locations also had an impact on the treatment response. The OS and CSS of the non-EGJ subgroup patients were significantly associated with pCRT, whereas the improvements in OS and CSS of GASC at the EGJ site by pCRT were not significant (**Figures 3C, D**). Additionally, the OS and CSS of patients with a tumor diameter greater than 5 cm (p <0.001), differentiation grade 3-4 (p = 0.008), pathological T3 - 4 (p <0.001), pathological N2 - 3 (p <0.001), and pathological TNM III-IV (p <0.001) were significantly associated with pCRT (**Figure 3E-N**). However, the OS and CSS of patients with a tumor diameter ≤ 5 cm, pathological T stage 1-2, pathological N stage 0-1, and TNM I-II stage did not show a significant association with pCRT (**Supplementary Figures S3C–L**).

**Figure 4:**
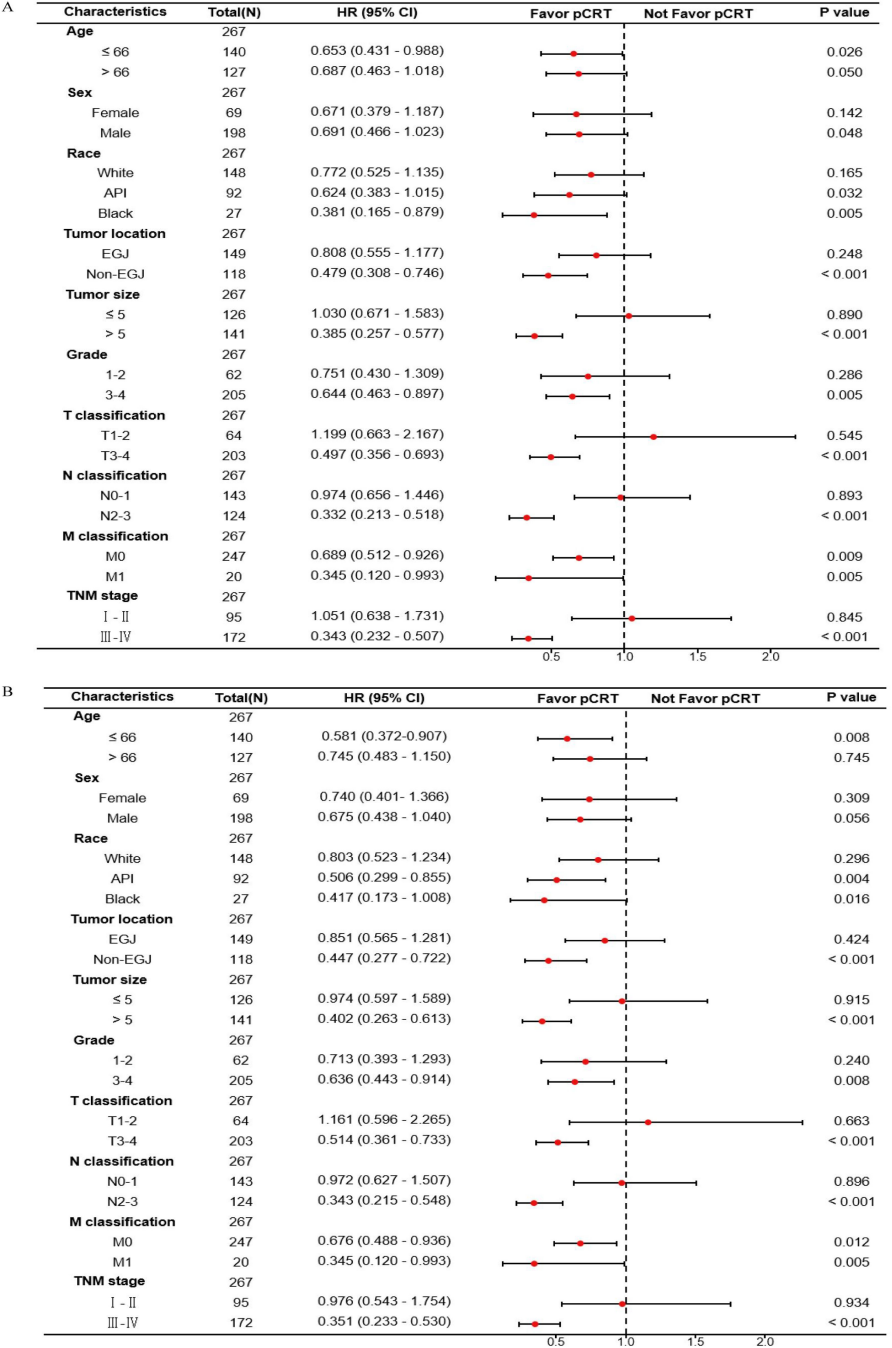
Forest plot of the association of pCRT with median overall survival **(A)** and median cancer-specific survival **(B)** in subgroup analyses.

Furthermore, the HR values of pCRT patients compared to non-pCRT patients in each variable subgroup were obtained, and forest plots related to the median OS and median CSS were drawn. It could be seen that the mOS in subgroups aged ≤ 66, Black, non-EGJ, tumor > 5 cm, tumor differentiation grade 3-4, T3-4 stage, N2-3, and TNM III-IV was significantly associated with perioperative chemoradiotherapy (**Figure 4A**; **Supplementary Table S2**). Similarly, the CSS in subgroups aged ≤ 66, non-EGJ, tumor > 5 cm, tumor differentiation degree 3-4, T3-4 stage, N2-3, and TNM III-IV was also significantly associated with perioperative chemoradiotherapy (**Figure 4B**; **Supplementary Table S3**). However, the CSS of the API subgroup, but not the Black and White subgroups, was significantly associated with perioperative chemoradiotherapy. In summary, this indicates that the GASC population aged ≤ 66 years, non-EGJ, tumor > 5 cm, tumor differentiation degree 3-4, T3-4 stage, N2-3, and TNM III-IV could significantly benefit from perioperative chemoradiotherapy.

In addition, a stratified analysis was carried out in the pCRT group to assess the role of radiotherapy in GASC treatment by comparing the survival prognosis of GASC subgroups with perioperative chemotherapy combined with radiotherapy (both) versus those with perioperative chemotherapy alone (alone). In this study, 83 patients received perioperative chemotherapy combined with radiotherapy and 53 patients received perioperative chemotherapy alone. Among the combined treatment subgroup, 52 patients underwent radiotherapy preoperatively, 29 postoperatively, and 2 in both periods. The combined chemoradiotherapy group showed significantly improved OS and CSS of GASC compared to the chemotherapy alone group (p = 0.008, HR = 0.598, 95%CI 0.389 - 0.917 for OS; p = 0.002, HR = 0.534, 95%CI 0.337 - 0.847 for CSS) (**Supplementary Figure S4**). These findings imply that radiotherapy may have a significant impact on enhancing the long-term prognosis of patients with GASC.

## Discussion

Gastric adenosquamous carcinoma (GASC) is a rare malignant entity characterized by the simultaneous coexistence of adenocarcinoma and squamous cell carcinoma components (21, 22). The majority of studies have proposed that the malignancy of GASC is greater than that of GAC, yet the underlying etiological mechanism has not been elucidated (2, 6, 23, 24). Currently, several hypotheses have been proposed to speculate on the origin of the squamous cell carcinoma component of GASC, which might be beneficial for understanding its pathogenesis. For instance, squamous cell carcinoma component may derived from squamous metaplasia of adenocarcinoma, carcinogenesis of ectopic squamous epithelium or differentiation of stem cells with bidirectional differentiation potential into glandular and squamous cells (2, 21, 22, 25–27). The diagnosis of gastric adenosquamous carcinoma (GASC) is primarily established through histopathological examination of biopsy specimens, with definitive confirmation often achieved following surgical resection and subsequent immunohistochemical analysis. Notably, there are no standardized treatment guidelines specifically for GASC in current clinical practice. Consequently, the management of GASC generally follows a therapeutic strategy analogous to that of GAC, centered around surgical resection as the primary modality, supplemented by multimodal treatment approaches including chemotherapy, radiotherapy, or immunotherapy, as clinically indicated.

Over the past few decades, with the implementation of D2 radical gastrectomy, researchers have recognized that relying solely on surgery can no longer significantly improve the long-term prognosis of patients with gastric cancer. The ACTS-GC study and CLASSIC study have demonstrated that postoperative adjuvant chemotherapy can significantly prolong the overall survival time of patients with gastric adenocarcinoma compared to surgery alone (28, 29). The MAGIC trial, a landmark study, established the efficacy of perioperative therapy in gastric adenocarcinoma (30). Consequently, the perioperative treatment paradigm - comprising neoadjuvant treatment + surgical resection + postoperative adjuvant therapy - has become a standard approach for locally advanced gastric adenocarcinoma. However, due to the rarity of GASC, prospective clinical trials evaluating perioperative CRT remain scarce, leaving its long-term prognostic impact uncertain. Available evidence is limited and conflicting. Ebi M et al. reported a case of long-term relapse-free survival (RFS) in a GASC patient receiving S-1 monotherapy postoperatively (31). Ge Y et al. observed improved overall survival (OS) and cancer-specific survival (CSS) in GASC patients receiving chemotherapy, based on a SEER database analysis (2). Conversely, a retrospective study that enrolled 76 cases of GASC recently identified that adjuvant therapy did not improve survival time (p = 0.266, HR= 0.394, 95%CI, 0.077–2.030) (20). Li HS et al. similarly concluded that chemotherapy’s role in resected GASC remains unclear (3). Akce M et al., analyzing the National Cancer Database (NCDB), reported CRT utilization rates of 14.1% (chemotherapy) and 37.6% (radiotherapy) in GASC patients, yet GASC survival outcomes continued to lag behind GAC (9). To address this knowledge gap, our study provides the first comparative analysis of long-term outcomes in GASC patients with versus without perioperative CRT, offering preliminary evidence to guide clinical decision-making.

Leveraging the SEER database and our institutional gastric cancer database, this study evaluated survival outcome in GASC patients through robust statistical analysis of a relatively large cohort. We demonstrated that perioperative radiochemotherapy significantly improves long-term survival, particularly in patients with biologically aggressive disease—defined by tumors >5 cm, poorly differentiated/undifferentiated histology, or advanced TNM staging. Subgroup analysis validated that combined modality therapy (radiotherapy plus chemotherapy) confers superior survival benefits over chemotherapy alone, indicating that radiotherapy plays a critical role in enhancing locoregional tumor control. These findings establish a foundation for stratified treatment protocols emphasizing risk-adapted radiotherapy incorporation in high-risk GASC subgroups. In clinical practice, extensive research has established similarly that the combination of radiotherapy, surgery, and chemotherapy yields remarkable efficacy in squamous cell carcinoma treatment, such as locoregionally advanced head and neck squamous cell carcinoma (HNSCC) (32), locally advanced cervical cancer (33) and esophageal squamous cell carcinoma (34). The therapeutic efficacy of radiotherapy in squamous cell carcinomas may stems from their biological characteristics: high mitotic indices reflecting rapid cellular proliferation and enhanced susceptibility to DNA damage-induced cell death (35, 36). In the future, research on the mechanisms by which perioperative chemoradiotherapy benefits patients with GASC should explore the following aspects: 1. The differences in radiosensitivity between the adenocarcinoma and squamous cell components; 2. The molecular pathways mediating this radiosensitivity; 3. The optimal radiotherapy sequence within multimodal treatment regimens.

Current gastric cancer treatment guidelines lack specific recommendations for perioperative chemotherapy regimens in GASC, necessitating the adoption of regimens established for gastric adenocarcinoma. The RESOLVE trial established that the SOX regimen (S-1 plus oxaliplatin) significantly improves 5-year disease-free survival (DFS) and overall survival (OS) when administered perioperatively to patients with locally advanced gastric cancer (37). Similarly, the PRODIGY study demonstrated that preoperative DOS (docetaxel, oxaliplatin, S-1) followed by postoperative S-1 monotherapy improves 3-year DFS compared to upfront surgery or adjuvant S-1 alone in this setting (38). In addition, the MATCH study further suggested superiority of the DOS regimen, achieving superior Major pathological remission (MPR) rates (25.45% vs 11.8% for DOS vs SOX) and 3-year progression-free survival (PFS) (52.3% vs 35%), positioning it as a potentially optimal neoadjuvant option (39). The FLOT4-AIO trial established the FLOT regimen (fluorouracil, leucovorin, oxaliplatin, docetaxel) as a new standard, significantly improving 3-year OS and DFS compared to ECF/ECX while achieving superior pathological remission rates (40). Notably, several retrospective studies regarding taxane-based regimens have shown promise in gastric cancer, though associated toxicities require careful management (41–43). Clinical evidence specific to GASC remains limited. Retrospective analyses have shown inconsistent results: Saito and Shin reported potential resistance to fluorouracil/platinum combinations in GASC (23), while other studies suggested survival benefits with paclitaxel/S-1 combinations in advanced cases (44). The SEER database’s limitations preclude analysis of perioperative treatment effects in GASC, but our institutional data indicate preferential use of taxane-based regimens (27/46 patients), reflecting growing clinical adoption of this approach.

Furthermore, a study investigating into the immunoprofile of GASC have reported that 25.0% of patients harbor deficient mismatch repair (dMMR) status, while 75.0% exhibit a combined positive score (CPS) ≥1, with 33.3% achieving CPS ≥10 (45). These findings suggest potential efficacy of immune checkpoint inhibitors in this population; however, their real-world applicability remains uncertain. Validation through large-scale, population-based studies is essential to confirm both the precise prevalence of PD-L1 expression and the clinical efficacy of immunotherapy in GASC. For HER-2-positive tumors, trastuzumab represents a standard addition. Notably, no definitive evidence currently identifies which specific therapy regimen most robustly correlates with long-term survival outcomes in GASC. Moreover, comprehensive biomarker discovery remains critical to developing predictive tools for therapeutic response and prognosis of GASC. Future studies should prioritize identifying molecular signatures capable of stratifying patients for optimized treatment strategies.

## Strengths and limitations

The strength of this study lies in the fact that considering the current low incidence of GASC, a large population-based cohort was established. Moreover, this is the first study to investigate the impact of perioperative chemoradiotherapy on the long-term prognosis of patients with GASC. Nevertheless, this study also has several limitations. Firstly, as a retrospective study, although we attempted to minimize the impact of confounding factors, selection bias remained unavoidable. Secondly, GASC data in this study were partly obtained from the SEER database, which lacks variables such as the proportion of squamous and adenocarcinoma components, specific perioperative treatment regimens and cycles, corresponding tumor markers, tumor pathological regression responses, and tumor recurrence status. Therefore, multicenter and large-scale RCT studies are required in the future to deeply explore the role of perioperative chemoradiotherapy in GASC.

## Conclusions

This retrospective study verified that perioperative chemoradiotherapy can improve the long-term OS and CSS of patients with GASC. Subgroup analysis found that patients with aged ≤ 66 years with tumor differentiation grade 3-4, T3-4, N2-3, and TNM III-IV could gain significant benefits from perioperative chemoradiotherapy. Moreover, the study demonstrated that patients with GASC receiving combined radiotherapy and chemotherapy had superior OS and CSS compared to those receiving chemotherapy alone, implying the crucial role of radiotherapy. This study provides an excellent evidence-based medical reference for GASC treatment.

## Data Availability

The raw data supporting the conclusions of this article will be made available by the authors, without undue reservation.
