# Supplementary material for: Survival benefit of perioperative chemoradiotherapy in patients with resectable primary gastric adenosquamous carcinoma: a population-based cohort study
Source: Front Oncol. 2025 Jul 2;15:1540106. doi: 10.3389/fonc.2025.1540106 (PMC12263382; doi:10.3389/fonc.2025.1540106)
Supplement: Supplementary file 1 [file DataSheet1.docx]

**Supplementary information**

Table S1. Detailed perioperative chemotherapy regimens of GASC from the PLAGH dataset.

| Treatment  classification | Regimens | Details | Proportion |
| --- | --- | --- | --- |
| Fluoropyrimidines and platinum agents | SOX | S-1 + Oxaliplatin | 5/46 |
|  | XELOX | Capecitabine + Oxaliplatin | 6/46 |
|  | FOLFOX | 5-Fluorouracil + Leucovorin + Oxaliplatin | 8/46 |
| Taxane-based combinations with either fluoropyrimidines or platinum agents | DOS | Docetaxel + Oxaliplatin + S-1 | 15/46 |
|  | FLOT4 | Fluorouracil + Leucovorin + Oxaliplatin + Docetaxel | 12/46 |

Table S2. Association of pCRT with total mortality in the subgroup analysis.

| **Characteristics** | **Overall Survival (OS)** | | | |
| --- | --- | --- | --- | --- |
|  | **Median OS (mo (IQR))** | | **Hazard ratio (95% CI)** | **P value** |
|  | **Non-pCRT group** | **pCRT group** |  |  |
| **Age** |  |  |  |  |
| ≤ 66 | 13 (6-50) | 27 (15-68) | 0.653 (0.431 - 0.988) | 0.026 |
| > 66 | 13 (4-29) | 21 (10-58) | 0.687 (0.463 - 1.018) | 0.050 |
| **Sex** |  |  |  |  |
| Female | 14 (5-88) | 26 (14-101) | 0.671 (0.379 - 1.187) | 0.142 |
| Male | 13 (6-30) | 26 (12-49) | 0.691 (0.466 - 1.023) | 0.048 |
| **Race** |  |  |  |  |
| White | 18 (6-51) | 26 (11-79) | 0.772 (0.525 - 1.135) | 0.165 |
| API | 13 (8-24) | 28 (19-36) | 0.624 (0.383 - 1.015) | 0.032 |
| Black | 5 (4-8) | 10 (8-38) | 0.381 (0.165 - 0.879) | 0.005 |
| **Tumor location** |  |  |  |  |
| EGJ | 18 (8-51) | 23 (13-68) | 0.808 (0.555 - 1.177) | 0.248 |
| Non-EGJ | 8 (5-19) | 26 (14-49) | 0.479 (0.308 - 0.746) | < 0.001 |
| **Tumor size** |  |  |  |  |
| ≤ 5 | 30 (12-150) | 33 (19-68) | 1.030 (0.671 - 1.583) | 0.890 |
| > 5 | 8 (4-14) | 19 (10-38) | 0.385 (0.257 - 0.577) | < 0.001 |
| **Grade** |  |  |  |  |
| 1-2 | 18 (7-30) | 26 (18-38) | 0.751 (0.430 - 1.309) | 0.286 |
| 3-4 | 12 (5-41) | 26 (11-66) | 0.644 (0.463 - 0.897) | 0.005 |
| **T classification** |  |  |  |  |
| T1-2 | 51 (12-N/A) | 38 (17-106) | 1.199 (0.663 - 2.167) | 0.545 |
| T3-4 | 10 (5-20) | 22 (12-38) | 0.497 (0.356 - 0.693) | < 0.001 |
| **N classification** |  |  |  |  |
| N0-1 | 23 (13-89) | 28 (13-79) | 0.974 (0.656 - 1.446) | 0.893 |
| N2-3 | 7 (4-11) | 20 (13-38) | 0.332 (0.213 - 0.518) | < 0.001 |
| **M classification** |  |  |  |  |
| M0 | 14 (5-46) | 27 (14-66) | 0.689 (0.512 - 0.926) | 0.009 |
| M1 | 6 (2-7) | 8 (7-15) | 0.345 (0.120 - 0.993) | 0.005 |
| **TNM stage** |  |  |  |  |
| Ⅰ-Ⅱ | 50 (19-150) | 38 (20-106) | 1.051 (0.638 - 1.731) | 0.845 |
| Ⅲ-Ⅳ | 8 (4-13) | 20 (11-36) | 0.343 (0.232 - 0.507) | < 0.001 |

Abbreviations: IQR, Interquartile Range; CI, Confidence Interval; API, Asian or Pacific Islander; EGJ, Esophagogastric Junction; TNM stage, Tumor-Nodes-Metastasis stage; Non-pCRT group, Non-perioperative Chemotherapy or Radiotherapy group; pCRT group, Perioperative Chemotherapy or Radiotherapy group; N/A, not applicable.

Table S3 Association of pCRT with cancer-specific mortality in the subgroup analysis.

| **Characteristics** | **Cancer Special Survival (CSS)** | | | |
| --- | --- | --- | --- | --- |
|  | **Median CSS (mo (IQR))** | | **Hazard ratio (95% CI)** | **P value** |
|  | **Non-pCRT group** | **pCRT group** |  |  |
| **Age** |  |  |  |  |
| ≤ 66 | 13 (6-50) | 29 (15-123) | 0.581 (0.372-0.907) | 0.008 |
| > 66 | 18 (6-51) | 21 (10-68) | 0.745 (0.483 - 1.150) | 0.745 |
| **Sex** |  |  |  |  |
| Female | 18 (5-91) | 26 (14-122) | 0.740 (0.401- 1.366) | 0.309 |
| Male | 14 (6-51) | 26 (12-68) | 0.675 (0.438 - 1.040) | 0.056 |
| **Race** |  |  |  |  |
| White | 20 (6-88) | 26 (11-132) | 0.803 (0.523 - 1.234) | 0.296 |
| API | 13 (8-24) | 28 (19-38) | 0.506 (0.299 - 0.855) | 0.004 |
| Black | 5 (4-12) | 10 (8-38) | 0.417 (0.173 - 1.008) | 0.016 |
| **Tumor location** |  |  |  |  |
| EGJ | 18 (9-88) | 26 (13-70) | 0.851 (0.565 - 1.281) | 0.424 |
| Non-EGJ | 8 (5-20) | 27 (14-123) | 0.447 (0.277 - 0.722) | < 0.001 |
| **Tumor size** |  |  |  |  |
| ≤ 5 | 51 (13-N/A) | 36 (19-N/A) | 0.974 (0.597 - 1.589) | 0.915 |
| > 5 | 8 (4-15) | 19 (10-58) | 0.402 (0.263 - 0.613) | < 0.001 |
| **Grade** |  |  |  |  |
| 1-2 | 19 (8-51) | 26 (19-38) | 0.713 (0.393 - 1.293) | 0.240 |
| 3-4 | 13 (5-59) | 26 (11-123) | 0.636 (0.443 - 0.914) | 0.008 |
| **T classification** |  |  |  |  |
| T1-2 | 88 (12-N/A) | 58 (17-148) | 1.161 (0.596 - 2.265) | 0.663 |
| T3-4 | 13 (5-21) | 23 (12-42) | 0.514 (0.361 - 0.733) | < 0.001 |
| **N classification** |  |  |  |  |
| N0-1 | 24 (13-N/A) | 28 (13-132) | 0.972 (0.627 - 1.507) | 0.896 |
| N2-3 | 8 (4-14) | 21 (13-38) | 0.343 (0.215 - 0.548) | < 0.001 |
| **M classification** |  |  |  |  |
| M0 | 16 (7-88) | 27 (14-123) | 0.676 (0.488 - 0.936) | 0.012 |
| M1 | 6 (2-7) | 8 (7-15) | 0.345 (0.120 - 0.993) | 0.005 |
| **TNM stage** |  |  |  |  |
| Ⅰ-Ⅱ | 59 (19-N/A) | 66 (21-N/A) | 0.976 (0.543 - 1.754) | 0.934 |
| Ⅲ-Ⅳ | 8 (4-14) | 21 (11-38) | 0.351 (0.233 - 0.530) | < 0.001 |

Abbreviations: IQR, Interquartile Range; CI, Confidence Interval; API, Asian or Pacific Islander; EGJ, Esophagogastric Junction; TNM stage, Tumor-Nodes-Metastasis stage; Non-pCRT group, Non-perioperative Chemotherapy or Radiotherapy group; pCRT group, Perioperative Chemotherapy or Radiotherapy group; N/A, not applicable.


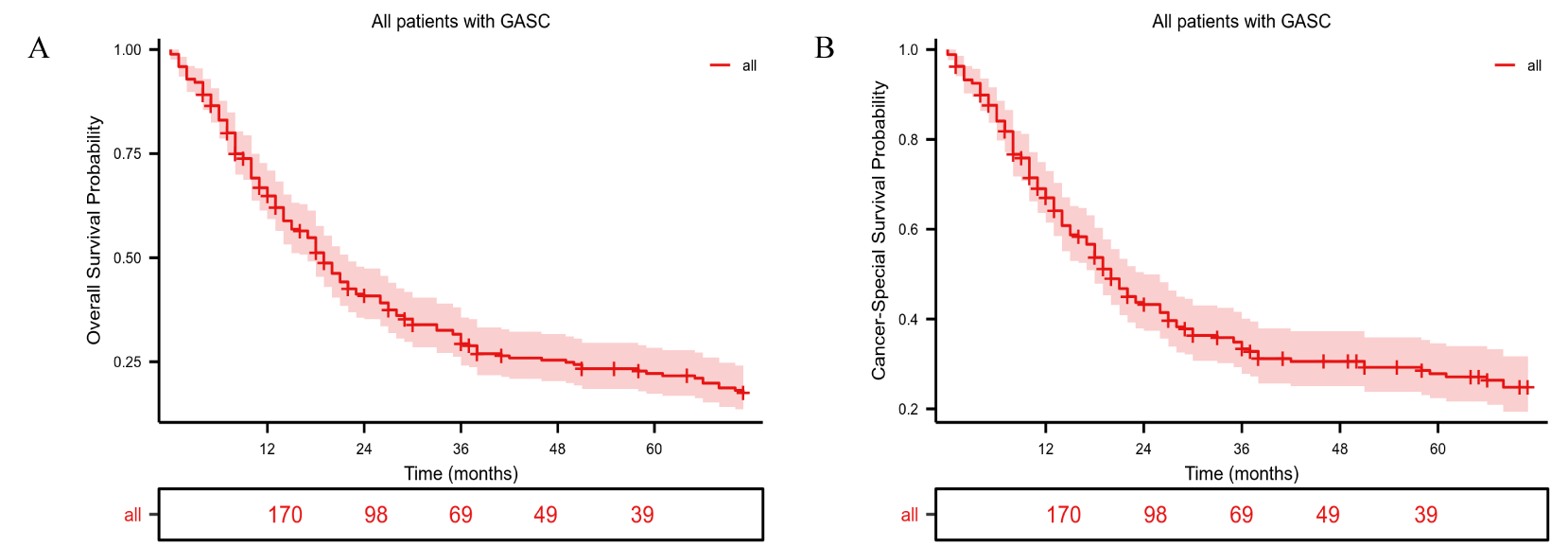


Figure S1. Kaplan-Meier survival curves of cumulative OS and CSS in all patients with gastric adenosquamous carcinoma included in this study. S1A, Kaplan-Meier survival curve of cumulative OS of GASC; S1B, Kaplan-Meier survival curve of cumulative CSS of GASC.


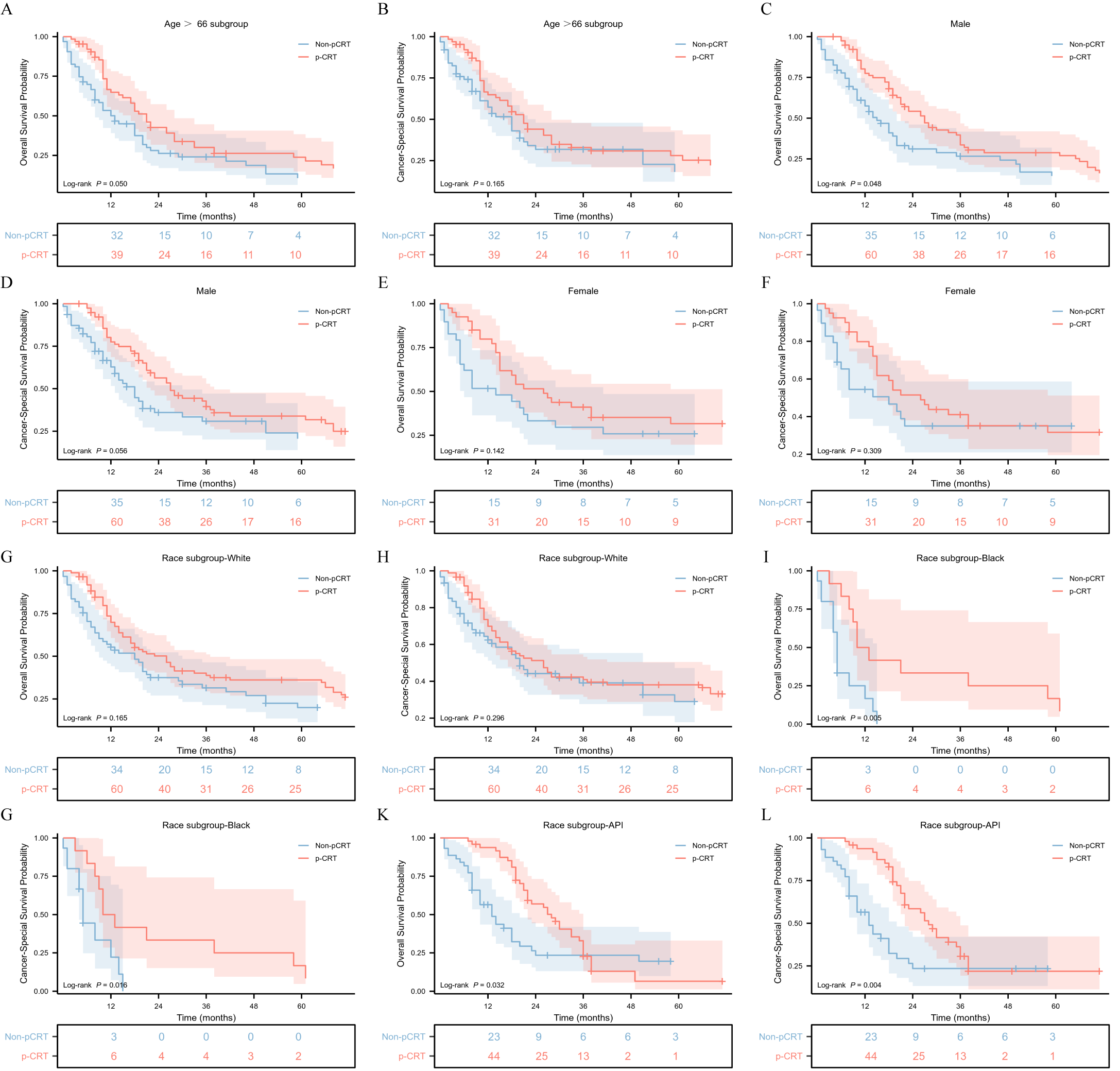


Figure S2. Kaplan-Meier survival curves of cumulative OS and CSS stratified by pCRT in different subgroups. Age > 66 years subgroup (A and B), male subgroup (C and D), female subgroup (E and F), white race subgroup (G and H), blacke race subgroup (I and J) and API race subgroup (K and L).


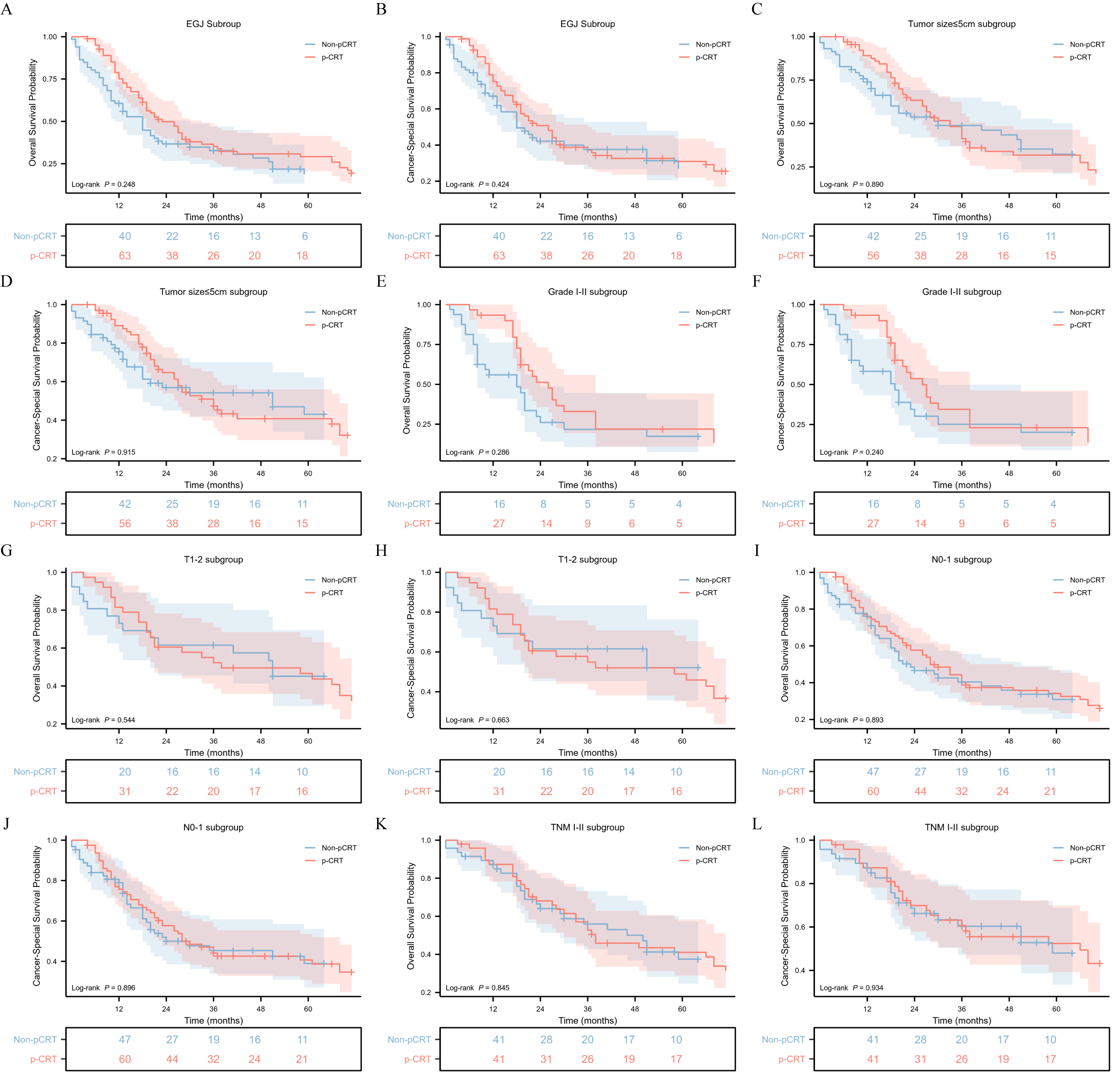


Figure S3. Kaplan-Meier survival curves of cumulative OS and CSS stratified by pCRT in different subgroups. EGJ subgroup (A and B), tumor size ≤ 5 cm subgroup (C and D), grade 1-2 subgroup (E and F), T 1-2 subgroup (G and H), N 0-1 subgroup (I and J) and TNM I-II subgroup (K and L).


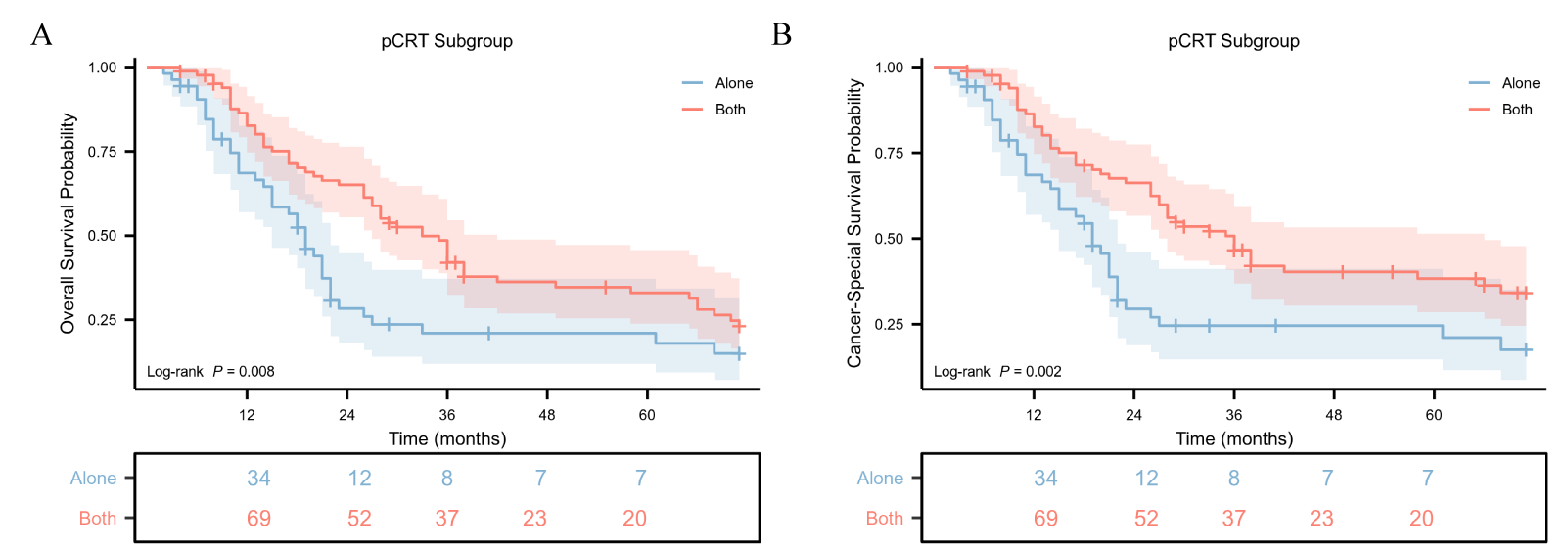


Figure S4. Kaplan-Meier survival curves of cumulative OS and CSS of perioperative chemotherapy combined with radiotherapy vs. perioperative chemotherapy alone in the pCRT subgroup. S4A, Kaplan-Meier survival curve of cumulative OS; S4B, Kaplan-Meier survival curve of cumulative CSS.
